# Influence of Dry Milling on Phase Transformation of Sepiolite upon Alkali Activation: Implications for Textural, Catalytic and Sorptive Properties

**DOI:** 10.3390/ma13183936

**Published:** 2020-09-05

**Authors:** Anna Walczyk, Robert Karcz, Joanna Kryściak-Czerwenka, Bogna D. Napruszewska, Dorota Duraczyńska, Alicja Michalik, Zbigniew Olejniczak, Anna Tomczyk, Agnieszka Klimek, Krzysztof Bahranowski, Ewa M. Serwicka

**Affiliations:** 1 Jerzy Haber Institute of Catalysis and Surface Chemistry, Polish Academy of Sciences, Niezapominajek 8, 30-239 Krakow, Poland; ncawalcz@cyf-kr.edu.pl (A.W.); nckarcz@cyf-kr.edu.pl (R.K.); nckrysci@cyf-kr.edu.pl (J.K.-C.); ncnaprus@cyf-kr.edu.pl (B.D.N.); ncduracz@cyf-kr.edu.pl (D.D.); ncmichal@cyf-kr.edu.pl (A.M.); 2 Institute of Nuclear Physics, Polish Academy of Sciences, Radzikowskiego 152, 31-342 Krakow, Poland; zbigniew.olejniczak@ifj.edu.pl; 3 AGH University of Science and Technology, Faculty of Geology, Geophysics and Environmental Protection, al. Mickiewicza 30, 30-059 Krakow, Poland; tomczyk.an@gmail.com (A.T.); agaklimek@o2.pl (A.K.)

**Keywords:** sepiolite, grinding, alkali activation, loughlinite, magnesium silicate hydrate, basicity, aldol condensation, Baeyer-Villiger oxidation, CO_2_ sorption

## Abstract

Activation of natural sepiolite by means of grinding in a planetary mill followed by wet NaOH activation was studied for the purpose of endowing the product with enhanced basicity for potential catalytic/sorptive applications. Synthesized solids were characterized with X-ray powder diffraction (XRD), N_2_ adsorption/desorption, scanning electron microscopy (SEM), energy dispersive (EDX), atomic absorption (AAS), Fourier-transform infrared (FTIR) and ^29^Si magic angle spinning nuclear magnetic resonance (MAS NMR) spectroscopies. Surface basicity was determined by titration with benzoic acid. Grinding changed the pathway of sepiolite phase transformation upon NaOH treatment. The as-received sepiolite evolved to Na-sepiolite (loughlinite) with a micropore system blocked by nanocrystalline Mg(OH)_2_, while ground samples yielded magnesium silicate hydrate phase (MSH), with well-developed microporous texture. In unmilled sepiolite desilication involved preferential leaching of Si from the center of the structural ribbons, while in ground samples additional loss of Si from ribbon-ribbon corner linkages was observed. In all cases treatment with NaOH led to enhancement of surface basicity. Synthesized materials were tested as catalysts in a base-catalyzed aldol self-condensation of acetone and oxidation of cyclohexanone to ε-caprolactone, as well as CO_2_ sorbents. Catalytic trends depended not only on samples’ basicity, but also on texture and phase composition of the catalysts. Grinding combined with alkali activation proved a simple and effective method for boosting CO_2_-sorption capacity of sepiolite to the level comparable to amine-functionalized, acid-activated sepiolite sorbents.

## 1. Introduction

Grinding of clay minerals is a very common treatment used both at the industrial scale and in research laboratories. Usually the primary goal is the reduction of particle size, but the treatments are known to induce significant changes in other physico-chemical properties of the mineral, by affecting its structure, texture and chemical reactivity [1,2]. Understanding of these phenomena is essential for different technological applications of clays, such as e.g., engineering of materials for adsorption and catalysis, preparation of mineral fillers, ceramic industry, agriculture, pharmacology, cosmetology, etc. Another type of modification of clay mineral is activation with acid or base, in order to selectively leach basic or acidic elements, respectively [3,4]. Such treatment modifies the mineral porosity by creating voids within the clay lattice, and endows the material with enhanced acid or base properties, factors of crucial importance for applications in catalysis and sorption. As shown in the recent review by Komadel [3], very many reports deal with acid-activated clays, with much fewer addressing the alkali-treated clays [4]. The latter, however, gain rapidly on importance, due their potential as base catalysts for valorization of biomass in the most common transesterification process [5], or as alkaline adsorbents favoring capture of acidic CO_2_ molecules [6]. Clays are cheap, abundant and environmentally benign materials, thus offer the possibility of developing low-cost, eco-friendly catalysts and adsorbents. Most of the research into basic clay-derived materials focuses on the alkali-modified montmorillonite, the main component of bentonite rocks, and the most widely investigated clay mineral [7,8,9,10]. However, we have recently pointed out that for the purpose of developing solids with basic functionality, magnesium-rich clay minerals, with stronger intrinsic basicity, such as e.g., sepiolite, are especially suited [11,12]. 

**Figure 1:**
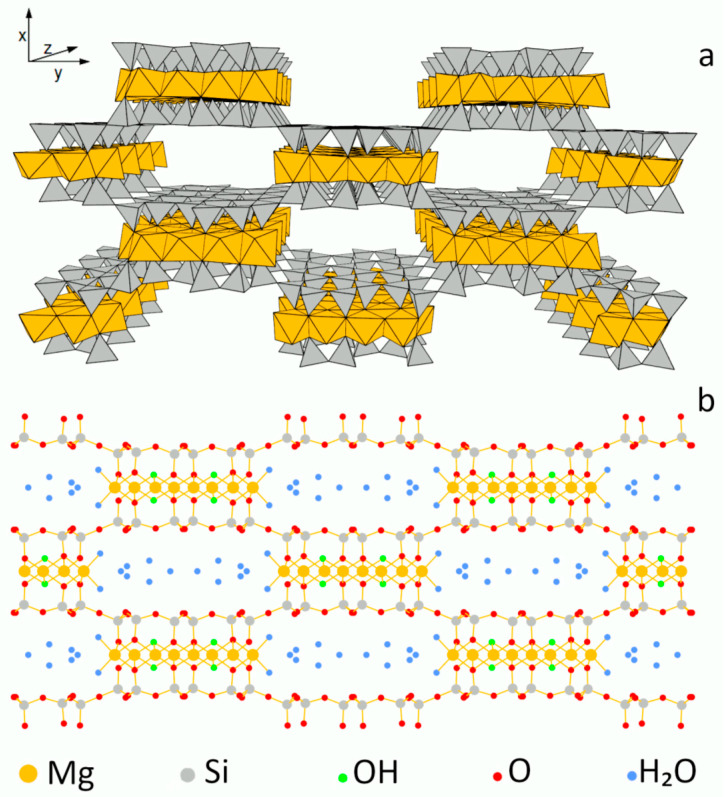
Model of sepiolite structure: (**a**) polyhedral, (**b**) ball and stick.

Sepiolite is a hydrated magnesium silicate with fibrous morphology, of theoretical unit cell formula Mg_8_(H_2_O)_4_(OH)_4_[Si_12_O_30_]·nH_2_O (n ≤ 8) [13]. The structure of sepiolite, shown in Figure 1, consists of 2:1 phyllosilicate ribbon-like blocks, corner-linked to each other by Si-O-Si bonds joining inverted SiO_4_ tetrahedra from neighboring ribbons. Such an arrangement results in structural tunnels with an effective cross-section of 10.6 × 3.7 Å [14,15,16,17], which are the reason for the microporosity and high specific surface area of sepiolite. Each terminal Mg site at the ribbon edge coordinates two water molecules, protruding into the tunnels. In addition, tunnels are filled with zeolitic water. Some Al(Fe) for Si substitution in the tetrahedral sheet, and Al(Fe) for Mg substitution in the octahedral sheet may occur in natural sepiolite and generate certain ion exchange capacity. 

We have shown that treatment of sepiolite with aqueous solution of NaOH is an easy tool for tailoring the materials’ basicity, and associated catalytic (acetone self-condensation, and cyclohexanone oxidation) and sorptive (CO_2_) properties [12]. Upon alkali activation a gradual transformation of sepiolite into partially desilicated Na-sepiolite (loughlinite) occurred, accompanied by intraporous formation of magnesium hydroxide nanoparticles. The latter was found to be the main factor responsible for the enhancement of basicity-related surface properties. 

Studies on Mg-bearing natural minerals and synthetic hydrotalcites have proved that mechanical grinding, when appropriately optimized, may also serve as means to enhance performance of basic catalysts, mainly due to the decrease of the catalyst crystal size [11,13]. Therefore, in the search for further means of improvement of the sepiolite catalytic/sorptive properties, the effect of grinding pretreatment in a planetary mill on the efficiency of the alkali activation of this mineral was investigated in the present work. Grinding of sepiolite has been addressed in several reports [18,19,20,21,22]. Generally, the treatment was found to result in the breakage of sepiolite fibers, loss of crystallinity and decrease of the specific surface area. Upon long-grinding a complete destruction of fiber morphology and structure amorphization was observed. However, the joint operation of both treatments, i.e., alkali activation and grinding, as well as their effect on catalytic and sorptive properties of sepiolite, have not been, as yet, described in the literature. 

The focus of this study is the effect of combined alkali and grinding pretreatment on the evolution of sepiolite structural and surface properties, in particular basicity, important for potential applications in catalysis and sorption. Base-catalyzed aldol self-condensation of acetone and oxidation of cyclohexanone to ε-caprolactone were chosen as test reactions. CO_2_ uptake, favored by the adsorbent alkalinity, was also investigated. Results were referred to the performance of an untreated sepiolite.

## 2. Materials and Methods 

### 2.1. Materials

Commercial sepiolite provided by Tolsa SA (Madrid, Spain), referred to as Sep, was used as the parent material. Grinding of sepiolite was carried out with aid of the planetary mono mill PULVERISETTE 6 (Fritsch, Idar-Oberstein, Germany) (600 rpm, with 4 g sample batches, using twenty-four 10-mm-diameter silicon nitride balls). The applied grinding times were 10, 30 and 60 min. For each grinding time, a new portion of the parent sepiolite was used. The millings lasting ≥ 10 min were performed in 15 min intervals, with a 10-min break between the grinding periods. Wet alkali activation consisted of treatment of sepiolite powder with a 2M NaOH (Sigma-Aldrich, Saint Louis, MO, USA, reagent grade) solution (1g/100 mL), upon vigorous mixing, at 90 °C, for 3 or 24 h. The recovered alkali-treated sepiolite samples were washed 5 times by dispersing in distilled water followed by centrifugation, and dried at 50 °C. The samples are denoted Sep/gr-time/NaOH-time, thus indicating the duration of grinding and the duration and temperature of alkaline treatment. For instance, sample signature Sep/gr-30’/NaOH-3h, means sepiolite ground for 30 minutes and treated with hot (90 °C) NaOH solution for 3 hours. In addition, a sample of brucite, Mg(OH)_2_, was prepared by precipitation at pH = 10 from aqueous solutions of Mg(NO_3_)_2_ and NaOH. All chemicals used in materials’ syntheses, adsorption and catalytic experiments were reagent grade, purchased from Sigma-Aldrich.

### 2.2. Methods of Physico-Chemical Characterization

Powder X-ray diffraction patterns were recorded with X’Pert PRO MPD (PANalytical, Almelo, The Netherlands) diffractometer, using Cu Kα radiation (40 kV, 30 mA), a flat graphite monochromator in the diffracted beam and a step size of 0.0334°. The setup included a programmable automated divergence slit giving a constant, illuminated sample length of 10 mm.

Scanning electron microscopy-energy dispersive X-ray spectroscopy (SEM/EDX) analysis was carried out with aid of JEOL JSM-7500F (JEOL, Tokyo, Japan) coupled with an INCA PentaFetx3 EDX (Oxford Instruments, Abingdon, UK) system. SEM images were recorded for the uncoated samples deposited on 200 mesh copper grids covered with carbon support film. 

The amount of Mg and Si leached to the solution upon alkali activation of sepiolite samples was determined by atomic absorption spectrometry method, using Thermo Scientific 3500 (Thermo Electron Manufacturing, Cambridge, UK) equipment. 

N_2_ adsorption/desorption at −196 °C was measured with an AUTOSORB 1 (Quantachrome, Boynton Beach, FL, USA) instrument. The samples were outgassed at 120 °C for 20 h. BET formalism was used for the calculation of specific surface areas, and a t-plot was used for the micropore surface area (S_micro_) and micropore volume (V_micro_) evaluation. The total pore volume (V_tot_) was determined from the amount of N_2_ adsorbed at p/p_0_ = 0.996. The mean pore diameter (D_av_) was calculated with the D_av_ = 4V_tot_/S_BET_ formula. 

Fourier transform infrared (FTIR) absorption spectra in middle infrared were recorded using transmission mode with a Nicolet 6700 (Thermo Scientific, Madison, WI, USA) spectrometer, in the 4000–400 cm^−1^ range. Samples were prepared as KBr pellets—64 scans at 2 cm^−1^ resolution were taken for each sample. 

The solid state ^29^Si MAS NMR spectra were measured on the APOLLO console (Tecmag Inc., Houston, TX, USA) at the magnetic field of 7.05 T provided by the 300 MHz/89 mm superconducting magnet (Magnex Scientific, Abingdon, UK). A Bruker HP-WB high-speed MAS probe equipped with the 4 mm zirconia rotor and KEL-F cap was used to spin the sample at 4 kHz. The resonance frequency was equal to 59.515 MHz, and a single 3 μs rf excitation pulse corresponding to a π/2 flipping angle was applied. The number of averages was 512, with the acquisition delay equal to 30 s. The frequency scale in ppm was referenced to tetramethylsilane (TMS).

Basicity of the materials was determined from the amount of benzoic acid (BA) adsorbed at room temperature (pKa = 4.19), as a mean of three experiments ± standard error. Briefly, 0.01 g of the sample was dispersed in 10 mL of BA solution in cyclohexane and mixed for 3 h. Then the solid was centrifuged and the concentration of BA remaining in the solution was determined by recording UV-vis absorption spectra with a Shimadzu UV 160A (Shimadzu, Kyoto, Japan) spectrophotometer, using quartz cells of 1 cm optical path.

### 2.3. Catalytic Testing

Aldol self-condensation of acetone to diacetone alcohol (DAA) was used as a model base-catalyzed reaction. The reaction was performed for 3 h in thermostated glass batch reactors using Radleys Carousel 6 parallel reaction station (Radleys, Shire Hill, Saffron Walden, Essex, UK) with a magnetic stirrer (500 rpm), at 40 °C, using 0.03 g of the as-received dried sample as a catalyst and 5 mL of acetone. It was found that beyond 500 rpm the agitation speed had no effect on the rate of reaction. The reaction mixture was analyzed by gas chromatography using a Thermo Trace GC Ultra instrument (Thermo Electron Corporation, Austin, TX, USA) fitted with a TR-5 column with a flame ionization detector. Yield of DAA was determined as a mean value of the product from three reaction runs performed at the same conditions, ± standard error. Samples were withdrawn from the reaction mixture after fixed time and analyzed as described.

Base-catalyzed, mild oxidation of cyclohexanone to ε-caprolactone in the recently described H_2_O_2_/nitrile/bicarbonate system [23] was carried out for 3 h at 70 °C in a glass reactor using Radleys Carousel 6 parallel reaction station (Radleys, Shire Hill, Saffron Walden, Essex, UK), with magnetic stirring (500 rpm), using 0.06 g of catalyst, 6 mmol of cyclohexanone, 50 mmol of 30% H_2_O_2_, 0.1 mmol of NaHCO_3_ and 100 mmol of acetonitrile solvent. Increasing the stirring speed beyond 500 rpm had no effect on the rate of reaction. The reaction mixture was analyzed by gas chromatography using a Thermo Trace GC Ultra (Thermo Electron Corporation, Austin, TX, USA) instrument fitted with a TR-5 capillary column and a flame ionization detector. Identification of by-products was performed using GC–MS analysis with the same GC equipped with a TR-5-MS capillary column and a DSQ II mass detector (Thermo Electron Corporation, Austin, TX, USA). Yield of ε-caprolactone was determined as a mean value of the product from three parallel runs performed at the same reaction conditions, ± standard error. Samples were withdrawn from the reaction mixture after a fixed time and analyzed as described.

### 2.4. Sorption of CO_2_

Carbon dioxide sorption capacity was determined from CO_2_ isotherms obtained with a Micromeritics ASAP 2020 (Micromeritics, Norcross, GA, USA) analyzer at 0°C, using ca. 300 mg of the sorbent, in a pressure range from 0.2 to 760 mm Hg. Prior to the measurements, samples were outgassed at 150°C for 12 h. To get an estimate of the standard error of the measurement the CO_2_ sorption on the parent sepiolite was conducted twice.

## 3. Results and Discussion

### 3.1. SEM Analysis

**Figure 2:**
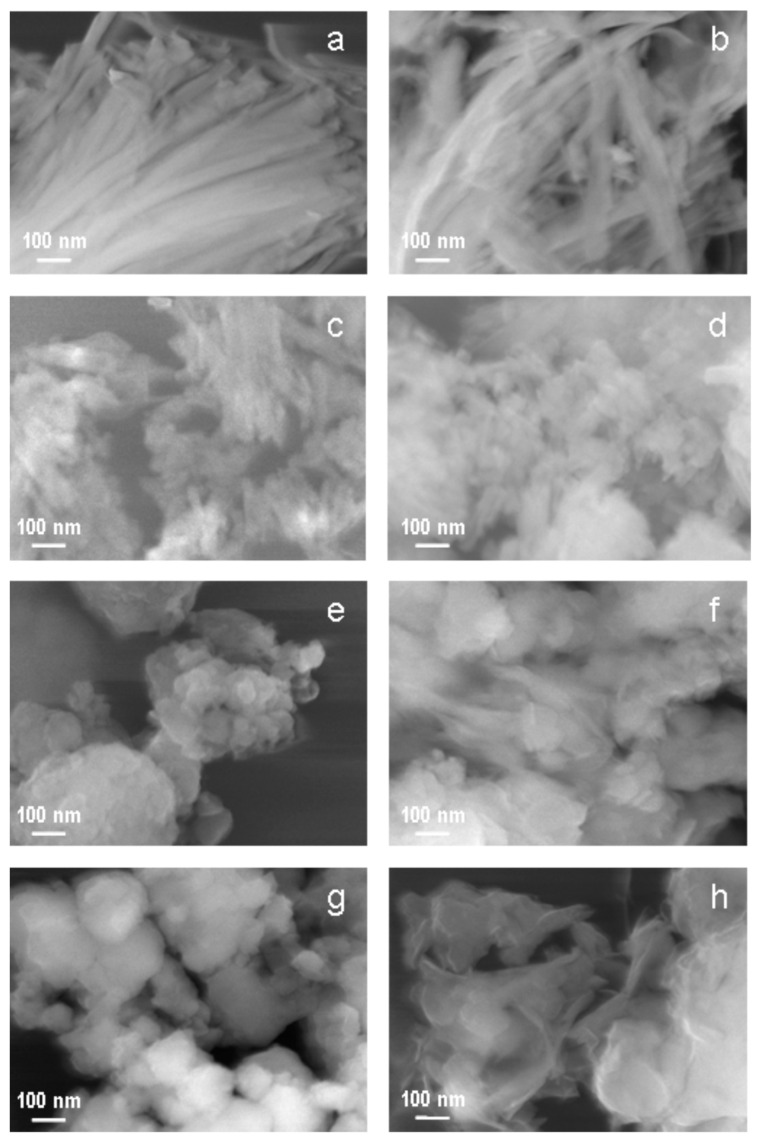
SEM images of: (**a**) Sep, (**b**) Sep/NaOH-24 h, (**c**) Sep/gr-10’, (**d**) Sep/gr-10’/NaOH-24 h, (**e**) Sep/gr-30’, (**f**) Sep/gr-30’/NaOH-24 h, (**g**) Sep/gr-60’, (**h**) Sep/gr-60’/NaOH-24 h.

The effect of grinding and wet alkali treatment on the morphology of sepiolite grains was studied with scanning electron microscopy and the micrographs are presented in Figure 2.

The parent sepiolite showed fibrous morphology typical for this mineral (Figure 2a). Grinding for 10 min resulted in breaking and disintegration of sepiolite fibers, but small individual particles still retained their elongated shape (Figure 2c). In samples subjected to longer grinding times (30 min —Figure 2e; 60 min—Figure 2g) the elongated grains disappeared completely. Instead, aggregates of irregularly shaped, rounded particles were observed. The effect of re-aggregation upon prolonged grinding of solids has been known for a long time [24]. In a ball mill, the particles get very close to one another due to mechanical pressure and friction, which eventually leads to the cold welding at the contact points.

Comparison of SEM images recorded for the parent and ground sepiolite samples with those obtained for the counterparts subjected to alkaline treatment shows that most pronounced changes in morphology occurred during alkali activation of sepiolite subjected to prolonged grinding. 

Thus, the parent sepiolite (Figure 2a) and the sample ground for 10 min (Figure 2c), after 24 h of treatment with NaOH solution at 90 °C, showed, generally, grains of quite similar morphology to that found in the starting materials, i.e., long fibers in the first case and much shorter, broken ones, in the other (Figure 2b,d, respectively). On the other hand, in materials ground for 30 and 60 min (Figure 2e,g, respectively), alkali treatment brought about a qualitative change in morphology. Thus, in both samples grinding lead to the complete destruction of fibrous particles and formation of spherical agglomerates, more compact for the longer the grinding time. Upon alkali treatment a partial disintegration of agglomerates accompanied by formation of very thin particles with laminar morphology was observed (Figure 2f,h, respectively), suggesting a deep change in the nature of the investigated silicate material. 

### 3.2. XRD Analysis

**Figure 3:**
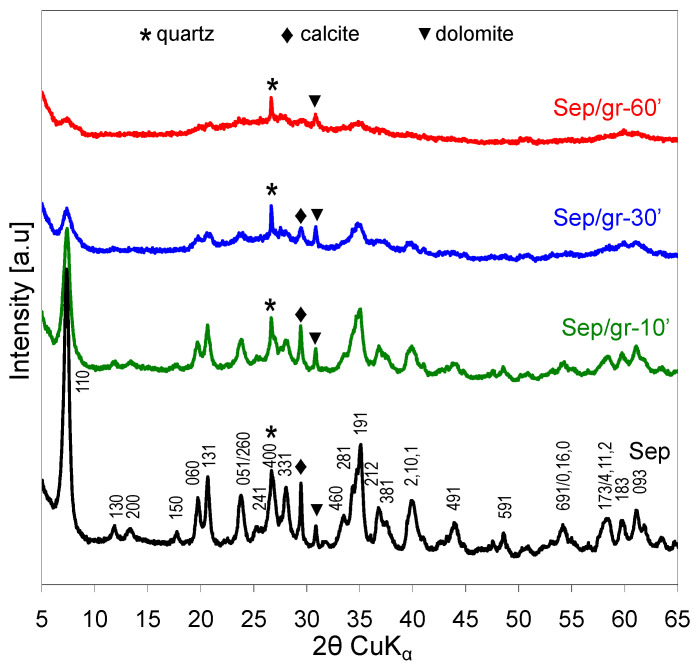
Effect of grinding on XRD patterns of sepiolite.

The parent material shows a characteristic X-ray diffraction pattern of sepiolite. The indexing, marked in Figure 3 (bottom), is based on the reported data (sepiolite, Ref. code 01-080-5781). Raw sepiolite also contains traces of quartz, calcite and dolomite impurities. The effect of grinding on the structure of sepiolite agreed, qualitatively, with the results reported by Cornejo and Hermosin [17]. As the grinding time increased, a gradual broadening of reflections belonging to sepiolite could be observed, attributable to the diminution of crystal size and growing crystal strain. The XRD pattern of the Sep/gr-60’ sample indicated that the material underwent strong amorphization, as the 110 reflection of sepiolite, as well as other most intense reflections were barely marked. The reflections belonging to quartz and dolomite were still visible, although the latter was clearly broadened, while crystalline calcite could not be detected any more. Thus, the susceptibility of sepiolite and its impurities to grinding reflected the general trend of the minerals’ hardness according to Mohs scale (sepiolite—2–2.5, calcite—3, dolomite—3.5–4, quartz—7) [25]. 

**Figure 4:**
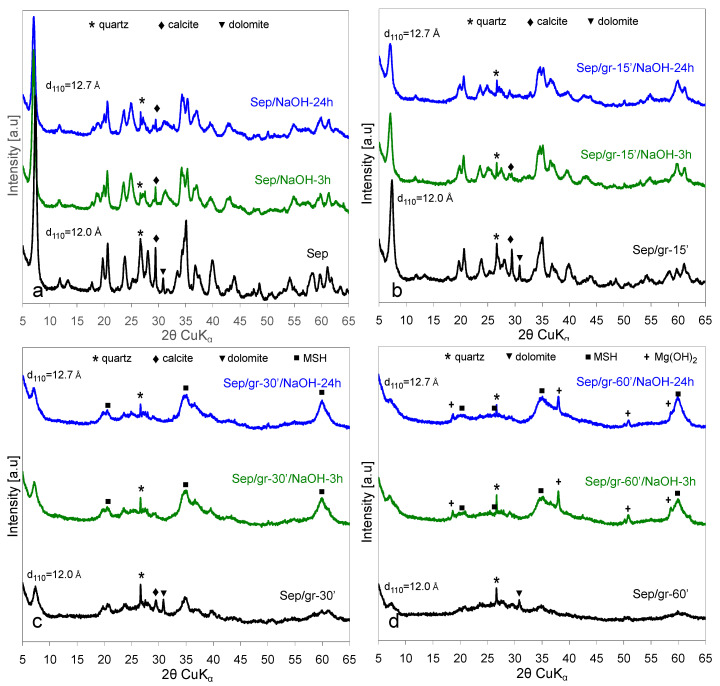
Effect of treatment with NaOH at 90 °C for 3 or 24 h: (**a**) sepiolite as-received, (**b**) sepiolite ground for 10 min, (**c**) sepiolite ground for 30 min, (**d**) sepiolite ground for 60 min.

Evolution of XRD patterns of the as-received and ground sepiolite samples upon treatment with NaOH at 90 °C for 3 or 24 h are illustrated in Figure 4. 

As reported earlier [12], in the case of an untreated sepiolite the observed changes pointed to the gradual transformation of sepiolite into its sodium form, loughlinite (Figure 4a). The structural modification was directly evidenced by the shift of the (110) reflection towards higher 2θ. The effect corresponded to the increase of d_110_ from 12.0 Å observed for the starting sepiolite to ca. 12.7 Å for the sample subjected to alkali treatment, the value characteristic of loughlinite (reference code 01-082-8023). The NaOH activation also affected the reflections of crystalline carbonate impurities. The most intense line of dolomite ceased to be visible and that of calcite diminished significantly. Additionally in the case of sepiolite ground for 10 min (Figure 4b), the main effect observed upon alkali treatment was the evolution of loughlinite structure. In addition, the reflections around 2θ = 60° appeared to gain on intensity in relation to the remaining peaks. The reason for the latter effect became clear after analysis of the impact the NaOH activation had on Sep/gr-30’ and the Sep/gr-60’ samples (Figure 4c,d, respectively). In both materials, features characteristic of a phase known as magnesium silicate hydrate (MSH) dominated the XRD patterns. MSH is a semi-amorphous phase of variable Mg/Si ratio, whose structure yields broad XRD peaks around 2θ values of 20°, 27°, 35° and 60°, first reported in a study of cement degradation by sea water [26]. MSH solids, which recently attracted attention as a promising, eco-friendly alternative to common, CaO-based Portland cement, as well as catalysts and adsorbents, are readily formed in the MgO-SiO_2_-H_2_O system, especially for the Mg/Si ratio higher than 0.5 [27,28,29,30,31,32]. Their structure is poorly defined and appears to be related to nanocrystalline turbostratic 2:1 magnesium phyllosilicates [33]. In the most strongly ground sepiolite sample Sep/gr-60’, alkali treatment also resulted in crystallization of Mg(OH)_2_, in addition to MSH (Figure 4d). In this material, only the small maximum at 12.7 Å could be related to the remnants of the sepiolite structure, obviously heavily destroyed by the joint grinding/alkali activation. Noteworthy, in addition to the 12.7 Å feature a weak broad hump evolved in this area, attributable to the layered MSH structure [33]. 

It should be noted that formation of Mg(OH)_2_, albeit amorphous to XRD analysis, was also observed upon alkali treatment of unground sepiolite, and resulted from formation of Mg(OH)_2_ nanoparticles within the sepiolite micropore system due to precipitation of Mg ions removed from the lattice upon formation of Na-sepiolite (loughlinite) [12]. Therefore, the product of wet NaOH activation of sepiolite has been identified as a composite of loughlinite and nano-Mg(OH)_2_. The appearance of brucite reflections in the XRD pattern of alkali-treated, most strongly ground sepiolite indicated that destruction of the parent clay lattice removed spatial constraints exerted by micropores and enabled crystallization of Mg(OH)_2_, so that the final product could be regarded as a composite of MSH and brucite, with possible traces of destroyed loughlinite structure.

### 3.3. Chemical Composition

The results of EDX analysis carried out for the investigated materials are shown in Table 1. Elemental composition of the parent sepiolite was in good agreement with the literature data for the Spanish sepiolite [34]. The only effect of grinding on the sepiolite composition was a small decrease of the amount of volatile component, pointing to a degree of sample dehydration. The relative proportions of the constituting elements remained practically constant, with the average Mg/Si ratio equal to 0.60. Treatment with NaOH solution strongly affected the composition of the recovered solids, the most pronounced effect being the fall of SiO_2_ content, in accordance with the known selective leaching of Si from sepiolite in the strongly basic environment [12,35]. The loss of Si during NaOH activation was enhanced by grinding. For selected samples the liquids remaining after alkaline activation were also subjected to chemical analysis, in order to assess the concentration of leached Si and Mg, and the data are presented in Table 1. The results show that the amount of Si extracted from the sepiolite lattice grew with time of the alkali activation and was strongly enhanced by the grinding pretreatment. Thus, while ca. 16% of Si was removed upon 24 h activation at 90 °C from the parent sepiolite, the amount of Si released from sepiolite ground for 1 h (Sep/gr-60’/NaOH-24 h) almost tripled. In contrast, the amount of leached Mg was much lower, and practically constant for all experiments. This effect, already described for alkali activation of sepiolite [12], was due to the fact that in the strongly alkaline environment any Mg^2+^ ions released from the sepiolite lattice upon contact with NaOH precipitated immediately as Mg(OH)_2_. In consequence, the concentration of Mg in the solution was determined by the solubility product of brucite. Accordingly, the Mg/Si atomic ratio in the NaOH-treated solids grew with respect to the value observed in the parent samples, both for the as-received sepiolite and for the ground materials, and could be maximized by extending the time of alkali activation and/or the grinding pretreatment. In the most strongly ground sample the degree of desilication was apparently too high to accommodate all Mg in the newly formed MSH, and formation of well crystalline Mg(OH)_2_ was observed. 

An increase of Na content was observed in the alkali-treated samples, the effect being most pronounced for the as-received sepiolite and for the Sep/gr-10’ sample subjected to the shortest grinding time (Table 1). In the latter samples the sepiolite lattice was either undamaged, or relatively little disturbed, and the effect could be assigned to the formation of Na-sepiolite (loughlinite), evidenced by XRD. In strongly ground Sep/gr-30’ and Sep/gr-60’, in which evolution of MSH phase was observed in XRD patterns, the uptake of sodium decreased, thus confirming that grinding-induced amorphization had a profound impact on the route of chemical transformation of sepiolite. 

### 3.4. FTIR Spectroscopy

**Figure 5:**
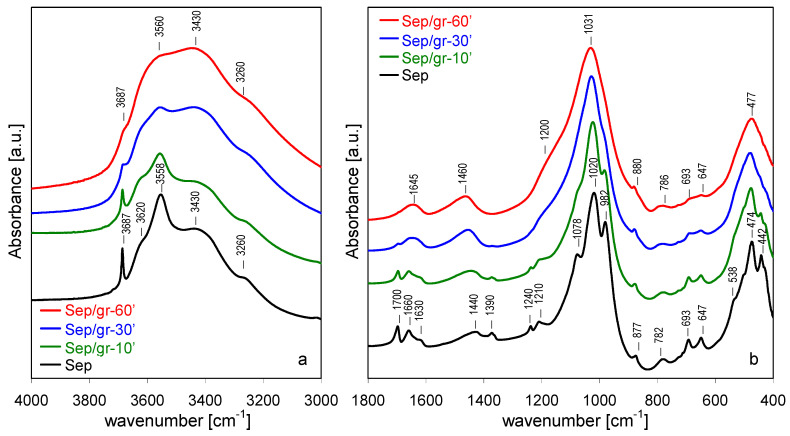
Evolution of FTIR spectra for sepiolite ground for different periods: (**a**) 3000–4000 cm^−1^, (**b**) 400–1800 cm^−1^.

FTIR spectroscopy proved to be a sensitive tool in determining the effects of various treatments on the bonding within the sepiolite structure [36,37,38,39,40,41,42,43,44]. Figure 5 shows the evolution of FTIR spectra upon increasing time of grinding in the 4000–3000 cm^−1^ range, where OH stretching modes are observed, and in the 1800–400 cm^−1^ region, in which water deformation and lattice modes appear. The assignments of bands follow our previous work and the references therein [12]. 

In the 4000–3000 cm^−1^ range (Figure 5a) sepiolite displays a sharp band at 3687 cm^−1^, corresponding to ν_OH_ stretching mode in Mg_3_OH units in the octahedral layer [37]. The broad bands at 3620 and 3558 cm^−1^ were assigned to OH stretching vibrations in H_2_O molecules coordinated to Mg at the ribbon edges [42], but it is possible that the former band could contain a contribution from OH stretches in dioctahedral defects associated with the presence of Fe and/or Al impurities cations in the octahedral layer [40,42]. The bands at 3430 and 3260 cm^−1^ were assigned to zeolitic water with, respectively, a lower and higher degree of hydrogen bonding [36,40]. 

In the 1800–400 cm^−1^ region (Figure 5b) three bands corresponding to OH bending mode were distinguished, with maxima around 1630, 1660, and 1700 cm^−1^, attributed to coordination water, and two types of zeolitic water with increasing strength of hydrogen bonding, respectively [36,37]. The bands at 1440 and 1390 cm^−1^ were due to carbonate impurities [45]. The strongest bands at 1020 and 982 cm^−1^ stemmed from Si-O in-plane stretching vibration, the 1078 cm^−1^ mode from out-of-plane Si-O stretches [42,44] and the 1210 cm^−1^ band was associated with the asymmetric stretching vibration of Si-O-Si linkages between the inverted tetrahedra at the joints of alternating ribbons [38]. The 877 cm^-1^ band was due to the deformation mode of carbonate impurities [46]. The 782 cm^−1^ band was attributed to O-Si-O and/or Si-O-Si bending vibrations [47], and the 693 and 647 cm^−1^ modes stemmed from translations and bends of hydroxyls in Mg_3_OH groupings, respectively [40,43]. The shoulder at 538 cm^−1^ corresponded to Mg-OH vibration and the band at 474 cm^−1^ was due to the Si-O-Si bends [44]. The band at 442 cm^−1^ was recently attributed to the deformation mode of MgO_6_ octahedral units, based on its response to NaOH activation [12] and by analogy to the spectrum of related mineral, palygorskite [47]. The low-intensity band at 1240 cm^−1^ was assigned to 5-member ring-like structural defects [12]. 

Grinding of sepiolite caused changes in FTIR spectra, similar to those reported previously [17]. In the range of OH stretching modes, the most pronounced line-shape alteration involved OH vibrations in structurally well-defined environments, i.e., the 3687 cm^−1^ band of Mg_3_OH groupings, and the 3620 and 3558 cm^−1^ bands due to H_2_O molecules coordinated to external Mg sites at the ribbon edges. The former, sharp and intense in the parent sepiolite, appeared only as a weak inflection in the most heavily ground sample. This broadening and loss of intensity is indicative of the growing disorder within the octahedral layer. The bands due to OH stretches in water attached to Mg also broadened considerably and lost intensity, while the absorption intensity in the area of broadened 3430 and 3260 cm^−1^ bands became distinctly higher. The effect was most likely related to increased contribution from water hydrogen bonded to surface silanols generated during lattice disruption due to prototropy, i.e., diffusion of protons from their original siting within the clay structure to recombine with broken bonds [48]. Spectra evolved also in the 1800–400 cm^−1^ range. The resolved OH bending modes, characteristic of various types of structural water, broadened and ultimately appeared as a single envelope around 1645 cm^−1^. Evolution of OH vibrational modes upon grinding of sepiolite has been attributed to the effect known as prototropy, which caused a transfer of some protons from the bound and/or zeolitic water to grinding-induced broken bonds in the amorphized sepiolite lattice, to form weakly bound hydroxyls [17]. Grinding also affected bonds within the silicate framework. This was evidenced by a significant broadening of Si-O stretching modes at 1210, 1078, 1020 and 982 cm^−1^, which reflected a growing structural disorder within the Si-O sublattice. Eventually, in the Sep/gr-60’ sample, the bands merged into a broad, single absorption centered at 1031 cm^−1^. Simultaneous evolution of a broad shoulder near 1200 cm^-1^ showed that degradation of the silicate sheets led to the formation of a certain amount of amorphous hydrous silica phase [49]. The process was likely facilitated by the occurrence of prototropy. All other sepiolite lattice modes, both those related to the tetrahedral (782, 474 cm^−1^) and to the octahedral sheets (693, 647, and 442 cm^−1^), were likewise significantly broadened by grinding, indicating that all associated lattice bondings were susceptible to destruction by the mechanochemical treatment. The carbonate stretching modes stemming from calcite and dolomite impurities also changed upon grinding, and yielded, eventually, a single, broad band around 1460 cm^−1^, the effect consistent with the structural degradation evidenced by XRD analysis. 

**Figure 6:**
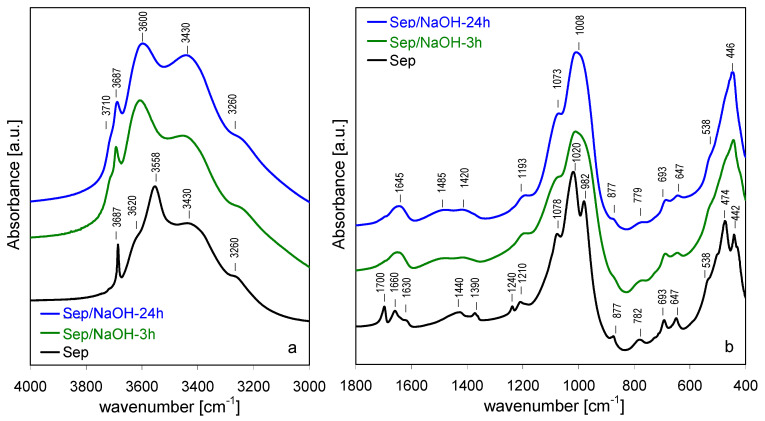
Evolution of FTIR spectra for sepiolite treated with NaOH at 90 °C, time effect: (**a**) 3000–4000 cm^−1^, (**b**) 400–1800 cm^−1^.

Upon treatment with NaOH solution a set of changes was observed in the FTIR spectra of treated solids, albeit of different character, depending on the degree of grinding pretreatment. As previously reported [12], in the case of the as-received sepiolite, the most important effects in the 3000–4000 cm^−1^ range were related to OH stretches in species interacting directly with Mg sites (Figure 6a). As alkali activation progressed, the band at 3687 cm^−1^, associated with the ν_OH_ stretching mode in Mg_3_OH entities in the octahedral layer, became increasingly broadened, while the bands related to water coordinated to Mg centers at the ribbon edges broadened and merged to yield a maximum at ca. 3600 cm^−1^. The changes reflected the fact that the nearest environment of Mg_3_OH units became less uniform as a result of structural damage caused by extraction of Si and/or removal of external Mg sites during alkaline activation and formation of Na-sepiolite. Moreover, as a result of NaOH treatment, a shoulder of growing intensity appeared around 3710 cm^−1^, attributed to magnesium hydroxide nanoclusters formed within the pore system of NaOH-activated sepiolite [12]. The positions of bands at 3260 and 3430 cm^−1^ corresponding to OH stretches in hydrogen bonded zeolitic water were not sensitive to alkaline activation, but there was an increase of their relative intensity, assigned to the effect of sodium for magnesium substitution, and to the increased contribution of H_2_O hydrogen bonded to surface silanols.

Analysis of 1800–400 cm^−1^ spectral range showed that NaOH treatment predominantly affected the bands related to vibrations involving Si sites, in accordance with the observed selective leaching of Si (Figure 6b). The increasing disorder within the silicate layers was reflected in a broadening of bands corresponding to stretching vibrations of Si-O (1210, 1078, 1020 and 982 cm^−1^) and O-Si-O and/or Si-O-Si bending modes (782 and 474 cm^−1^). In contrast, the bands involving translation and bending of hydroxyls in Mg_3_OH (693 cm^−1^ and the 647 cm^−1^) and deformation mode of MgO_6_ units (442 cm^−1^) were only slightly affected by the alkaline treatment, indicating that except for the partial loss of Mg from the ribbon edges due to the exchange with sodium cations, the octahedral layer retained its identity.

**Figure 7:**
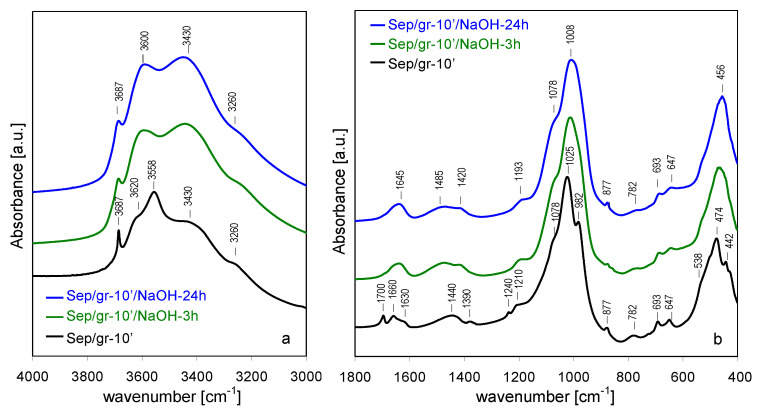
Evolution of FTIR spectra for sepiolite ground for 10 min and treated with NaOH at 90 °C, time effect: (**a**) 3000–4000 cm^−1^, (**b**) 400–1800 cm^−1^.

The effect of NaOH activation of the spectra of shortly ground sepiolite sample (Figure 7a,b) was quite similar to that observed for the parent sepiolite, in line with XRD data pointing to relatively small structural differences between Sep and Sep/gr-10’, and between their alkali-treated derivatives. However, a small but meaningful difference could be found in the OH stretching range, where neither in Sep/gr-10’/NaOH-3 h nor in Sep/gr-10’/NaOH-24 h a shoulder around 3710 cm^−1^ was observed, pointing to the absence of a significant amount of intraporous nanocrystalline Mg(OH)_2_ deposit. 

**Figure 8:**
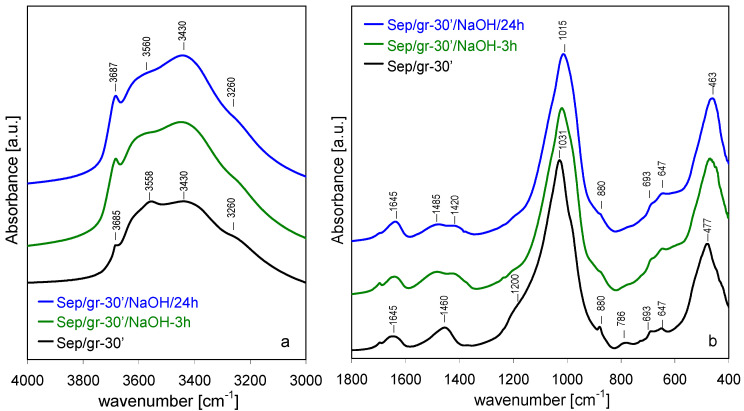
Evolution of FTIR spectra for sepiolite ground for 30 min and treated with NaOH at 90 °C, time effect: (**a**) 3000–4000 cm^−1^, (**b**) 400–1800 cm^−1^.

**Figure 9:**
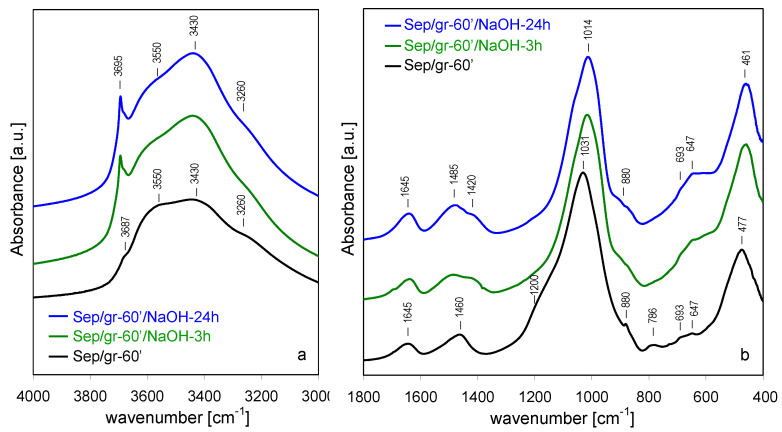
Evolution of FTIR spectra for sepiolite ground for 60 min and treated with NaOH at 90 °C, time effect: (**a**) 3000–4000 cm^−1^, (**b**) 400–1800 cm^−1^.

FTIR spectra of NaOH-treated Sep/gr-30’ and Sep/gr-60’ samples showed that the heavily ground materials responded differently to the alkali attack (Figure 8 and Figure 9). Although the spectra of starting materials were significantly broadened, several meaningful changes could be observed upon NaOH treatment. In the 3000–4000 cm^−1^ range the most striking effect was the increase of 3687 cm^−1^ band of ν_OH_ stretching mode in Mg_3_OH groupings, indicating that during alkali treatment of the amorphized material a reconstruction of the Mg-based sheet occurs (Figure 8a and Figure 9a). In addition, in the Sep/gr-60’/NaOH-24 h sample, a sharp band at 3695 cm^−1^, related to OH stretches in crystalline Mg(OH)_2_ appeared next to 3687 cm^−1^ one, in agreement with the XRD evidence pointing to the evolution of this phase in the most strongly ground material. Changes visible in the 400–1800 cm^−1^ range were consistent with the formation of layer-structured magnesium silicate hydrate phase, evidenced by XRD (Figure 8b and Figure 9b). Thus, the most intense Si-O stretching maximum shifted to 1014 cm^−1^, accompanied by the appearance of broad shoulders around 880 cm^−1^ and 680 cm^−1^, due to Si–OH vibrations and to libration modes of Mg_3_OH units, respectively, as expected for the evolution of MSH [50,51,52].

Noteworthy: the shoulder around 1200 cm^−1^, evidencing the presence of amorphous hydrous silica in the ground samples, disappeared after alkali treatment. This observation, combined with the fact that in the shortly ground sample there was no evidence of a shoulder at 3710 cm^−1^ characteristic of intraporous nanocrystalline Mg(OH)_2_, suggested that both phases were consumed in the process of MSH formation, as described by Walling et al. [51]. Only in the heavily ground sample, where Si-leaching resulted in Mg/Si ratio ≥ 1, the excess Mg crystallized as a separate, spatially unrestricted Mg(OH)_2_ phase, responsible for the appearance of the sharp 3695 cm^−1^ band.

### 3.5. ^29^Si MAS NMR Spectroscopy

**Figure 10:**
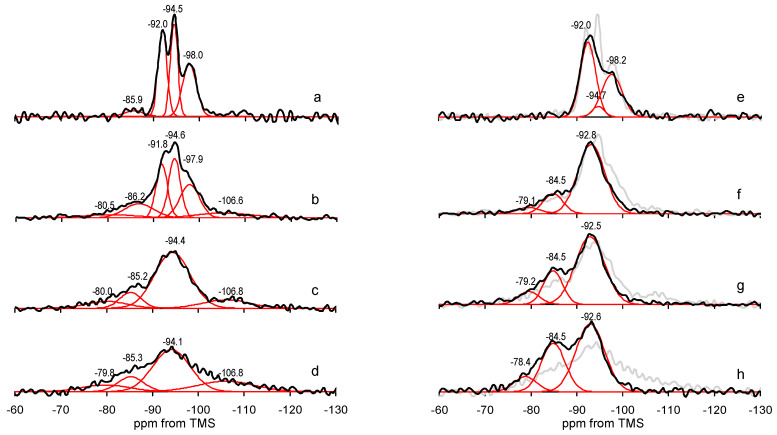
Evolution of ^29^Si MAS NMR of sepiolite samples upon grinding: (**a**) Sep, (**b**) Sep/gr-10’, (**c**) Sep/gr-30’, (**d**) Sep/gr-60’ and after treatment with NaOH at 90 °C for 24 h: (**e**) Sep/NaOH-24 h, (**f**) Sep/gr-10’/NaOH-24 h, (**g**) Sep/gr-30’/NaOH-24 h, (**h**) Sep/gr-60’/NaOH-24 h. Red lines represent deconvoluted spectrum components. For comparison, the spectra of samples before alkali treatment are shown in grey.

^29^Si MAS NMR spectroscopy enables insight into the structural details of silicate frameworks. In particular, the position of ^29^Si signal is sensitive to the degree of silicate lattice polymerization. In general, the sites with various connectivity are described as Q^n^, where Q denotes the Si center bonded to four oxygens and the superscript represents the number of other Si atoms bonded by oxygen bridges to Q site [53]. Thus, Q^0^ corresponds to monosilicates, Q^1^ to disilicates and chain end groups, Q^2^ to internal groups in chains, Q_3_ to chain branching sites and Q^4^ to the three-dimensional cross-linked framework. In the case of untreated sepiolite, whose structure is characterized by three crystallographically different Q^3^ Si positions (edge, near-edge and center of the ribbon, Figure 1), the ^29^Si MAS NMR spectrum consisted of three well-resolved resonances at −92.0, −94.5 and −98.0 ppm (Figure 10a), stemming from Si centers located in the near-edge, center and edge positions, respectively [54,55,56]. A weak resonance at −85.9 ppm was attributed to surface Q^2^ silanol groups [53]. Details of the spectra deconvolution are given in Table 2. 

In the spectrum of Sep/gr-10’ all three Q^3^ components were still visible, but their broadening reflected the occurrence of grinding-induced lattice disorder (Figure 10f). Partial destruction of the lattice was accompanied by the appearance of new resonances, at −80.0 and −106.6 ppm, attributed to Q^1^ and Q^4^ silicon environments, respectively. Emergence of Q^1^ and increase of Q^2^ indicated that milling caused a degree of depolymerization of the silicate framework, while the growth of Q^4^ evidenced formation of amorphous silica matter. ^29^Si MAS NMR spectra of Sep/gr-30’ and Sep/gr-60’ reflected growing degradation of the sepiolite lattice (Figure 10g,h). The initial Q^3^ resonances were no longer resolved and appeared as one broad signal around −94 ppm, and the share of Q^1^, Q^2^ and Q^4^ absorptions increased, in accordance with the XRD data evidencing almost complete disappearance of reflections characteristic of ordered sepiolite lattice. 

The effect of alkali activation depended critically on the mineral pretreatment (Figure 10e–h). In accordance with previous findings [12], in the case of parent sepiolite, there was no visible shift of the overall resonance position, but a significant decrease of the −94.5 ppm component, associated with Si sites at the center of structural ribbons, was observed (Figure 10e). The effect indicated that in the untreated sepiolite Si is preferentially leached from the middle of the tetrahedral sheets.

The response of ground sepiolite to NaOH activation was different. In the shortly ground sample, in addition to the decrease of the −94.5 ppm component, also the −98.2 ppm resonance, stemming from Si linking the neighboring ribbons, diminished. This suggests that the initial damage to the structure caused by milling involved breaking of the ribbon-ribbon linkages, rendering the Si centers in broken bonds more susceptible to alkali activation. The alkali treatment removed the traces of amorphous silica present in the Sep/gr-10’ sample, as evidenced by the disappearance of −106.6 ppm component, and enhanced the share of depolymerized species responsible for Q^1^ and Q^2^ resonances at −79.1 and −84.5 ppm. Changes observed in the spectra of Sep/gr-30’ and Sep/gr-60’ were of similar character, i.e., the broad Q^3^ resonance shifted upfield to ca. −92.5, pointing to the loss of Si from the center and edge positions in the remnants of sepiolite framework, the Q^4^ component disappeared indicating dissolution of the amorphous silica, and the contribution of the Q^1^ and Q^2^ resonances increased. The overall line-shape of Sep/gr-30’/NaOH-24 h and Sep/gr-60’/NaOH-24 h spectra, as well as positions and relative intensities of Q^1^, Q^2^ and Q^3^ components resembled those of MSH gels obtained from the Mg(OH)_2_-SiO_2_-H_2_O mixtures with Mg/Si = 1 [51]. The result was consistent with the XRD data pointing to formation of MSH. As previously reported, the Q^1^ and Q^2^ sites in MSH correspond to silanol species of Si–O–Si*–OH and (Si–O)_2_–Si*–OH sites, respectively [27,30], which agrees with detection of silanol bands in FTIR spectra of these samples.

In summary, ^29^Si MAS NMR analysis revealed a major difference between the as-received and the ground sepiolite with respect to alkali treatment. While NaOH activation of Sep sample led to the preferential leaching of Si located centrally in the structural ribbons, in ground samples a facile loss of Si from edge positions was also observed, suggesting that ribbon-ribbon linkages were particularly prone to milling-induced structural damage. Moreover, the spectra of heavily ground, NaOH-treated samples confirmed transformation of the solids into layered MSH materials.

### 3.6. Textural Properties and Surface Basicity in Relation to Catalysis and Sorption

Our previous report showed that wet alkali activation of sepiolite strongly influenced its textural properties and surface basicity [12]. In particular, the gradual transformation of sepiolite into partially desilicated loughlinite, accompanied by intraporous formation of magnesium hydroxide nanoparticles, resulted, on one hand, in the increase of surface basicity, but on the other, caused a blocking of micropores and a fall of specific surface area. Both phenomena affected the outcome of catalytic (acetone self-condensation, cyclohexanone oxidation) and sorption (CO_2_) experiments.

Textural characteristics data for materials investigated in the present work, obtained from the N_2_ adsorption/desorption isotherms, are summarized in Table 3. Grinding brought about a decrease of the specific surface area and q collapse of pore volume of sepiolite, the more pronounced, the longer the treatment (Sep, Sep/gr-10’, Sep/gr-30’, Sep/gr-60’). In general, particle diminution is expected to increase the specific surface area, but in the case of porous materials, such as sepiolite, the internal pore network may be destroyed and/or blocked by the amorphous phase produced during treatment, leading to the fall of textural parameters [17,18,19,20,21]. In addition, in heavily ground samples decrease of the specific surface may be due to the cold-welding effect [24], responsible for the formation of agglomerates observed in SEM study. After the most intense milling, the specific surface area and microporosity of Sep/gr-60’decreased by an order of magnitude, and the total pore volume became ca. 5 × lower in comparison with the as-received Sep sample. 

Analysis of textural data for NaOH-activated samples showed that impact of alkali treatment on the texture of the as-received sepiolite differed significantly from the effect it had on ground samples. Thus, in the case of unground mineral, a significant loss of the specific surface area occurred, mainly on the account of a dramatic reduction of sample microporosity, as both the micropore surface and the micropore volume fell by an order of magnitude (samples Sep, Sep/NaOH-3 h, Sep/NaOH-24 h). Elimination of micropores was paralleled by an increase of the average pore diameter. As reported previously [12], the effect was due to blocking of the micropore system by Mg(OH)_2_ nanoparticles formed as a result of instantaneous precipitation of Mg ions released upon formation of Na-sepiolite. A slight increase of the specific surface area and pore volume observed in the Sep/NaOH-24 h sample subjected to the most severe treatment possibly reflected the progressing corrosion of tetrahedral silica layers due to the abrasive action of alkali solution. 

**Figure 11:**
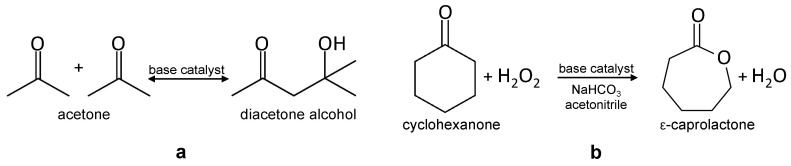
(**a**) Aldol self-condensation of acetone, (**b**) Bayer-Villiger oxidation of cyclohexanone to ε-caprolactone with H_2_O_2_ as an oxidant.

In contrast, in the case of ground sepiolite treated with NaOH, the specific surface area and porosity increased significantly. Comparison of textural data obtained for the shortly ground Sep/gr-10’ sample, with those of the parent sepiolite, showed that behavior of both materials in the alkaline environment was very different, despite close structural resemblance visible in XRD. In particular, the effect of almost complete blockage of microporosity in NaOH-treated Sep, caused by precipitation of Mg(OH)_2_ nanoparticles, was absent in alkali activates Sep/gr-10’, for which microporosity decreased to a much lesser degree. The phenomenon was consistent with results of FTIR data which did not detect the presence of nanocrystalline Mg(OH)_2_ deposit in NaOH-treated Sep/gr-10’ samples (lack of 3710 cm^−1^ shoulder in Figure 7a). Simultaneous disappearance of grinding-generated amorphous silica resonance in ^29^Si MAS NMR spectrum (Figure 11b) suggested that both phases were used up for the nucleation of quasi amorphous MSH [51]. Enhancement of textural parameters was particularly spectacular for samples subjected to stronger grinding pretreatment (Sep/gr-30’, Sep/gr-60’), in which S_BET_ values exceeding 300 m^2^/g were observed. The effect was accompanied by an increase of microporosity and a related decrease of the average pore size. In view of the XRD data one may infer that the enhancement of textural properties was related to further build-up of MSH phase, known to display a high specific surface area and micro/mesoporous character [57]. 

In order to assess the total surface basicity of the investigated materials, the samples were subjected to titration with benzoic acid [58], and the data on the amount of acid probe required for surface neutralization, are presented in Table 4, both per unit mass and, in view of varying specific surface area, per unit surface. Short grinding caused a slight increase in basicity, attributed to uncovering of Mg sites at (110) surfaces exposed by breaking of sepiolite fibers perpendicular to lath axis (SEM image in Figure 2c). Further grinding led to lowering of basicity, when expressed per gram of the sample, but, due to the strong decrease of the specific surface area, the areal basicity steadily increased, in line with progressing fractioning of sepiolite fibers. 

In all cases treatment with NaOH led to enhancement of basicity. The strong increase of basicity in Sep/NaOH-3 h and Sep/NaOH-24 h with respect to the Sep sample was attributed to the formation of nanocrystalline Mg(OH)_2_ precipitate [12]. In the case of ground samples, the increase of areal basicity was less pronounced, which suggested a different nature of basic sites. Indeed, nanocrystalline Mg(OH)_2_ was undetectable in these samples, and the weaker basicity had to be related to the newly generated MSH phase and/or loughlinite present in Sep/gr-10’/NaOH-3 h and Sep/gr-10’/NaOH-24 h samples. The appearance of well crystalline Mg(OH)_2_ in NaOH-treated Sep/gr-60’ samples did not cause a significant change in the observed basicity trend. 

Base catalyzed reactions, i.e., liquid phase aldol self-condensation of acetone and liquid phase Baeyer-Villiger oxidation of cyclohexanone to ε-caprolactone were carried out in order to check how the modification of texture and surface basicity impacts the catalytic properties of the investigated samples. 

Aldol condensation consists in reaction of two molecules with carbonyl groups to give hydroxylketones or unsaturated ketones and plays an important role in biomass feedstock transformation [59,60]. Aldol self-condensation of acetone yields diacetone alcohol (DAA) as the primary product (Figure 11a). The catalysts obtained in the present work were used as-received, without any thermal pretreatment. DAA was the only product of the acetone self-condensation reaction. The data in Table 4 show that both for the as-received and for the ground sepiolite samples the yield of DAA increased after alkali activation. Surprisingly, the enhancement of self-condensation activity was more pronounced on catalysts derived from ground samples, despite their lower areal basicity. Effects of this type were observed in other catalytic systems, when the catalyst surface displayed bifunctional, acid-base properties, because aldol condensations may also be catalyzed by acid sites [61,62,63]. In particular, weakly acidic silanol groups were found particularly suitable for cooperatively catalyzing the aldol condensation [63]. The presence of silanols in NaOH-treated, ground sepiolite catalysts was evidenced by FTIR and ^29^Si MAS NMR data. In view of this, it is proposed that the superior aldol condensation activity of catalysts derived from ground samples was due to the formation of MSH phase, enabling cooperative action of Mg-related basic sites and silanols. 

A different trend in catalytic activity was observed in the other test reaction, the Baeyer-Villiger oxidation of cyclohexanone to ε-caprolactone, the monomer used for manufacturing of biodegradable polymers [64]. Currently employed industrial processes are based on the stoichiometric reaction and use explosive organic peroxides, thereby generating copious amount of acid waste. Catalytic reaction with use of H_2_O_2_ as an oxidant represents an eco-friendly alternative [65] (Figure 11b). Addition of bicarbonate has a neutralizing effect on the reaction medium acidified by the presence of H_2_O_2_, which is beneficial for the basic catalyst, and provides an additional route of hydrogen peroxide activation [66], thus enhancing the reaction yield [23]. The reaction proceeded with ca. 70% selectivity over ground sepiolite samples, and with ≥ 90% selectivity over all remaining catalysts. According to the GC–MS analysis of the reaction mixture the nonselective products included cyclohexanone oxime and nitrocyclohexane, as well as several unidentified substances with much longer retention times, possibly corresponding to some polymerized products. The appearance of nitrogen-containing products pointed to the participation of the acetonitrile solvent in the nonselective reaction routes. The data on the ε-caprolactone yield gathered in Table 4 show that, in general, ground catalysts and their NaOH-treated derivatives performed poorer than the as-received sepiolite and the catalysts obtained by its alkali activation. Although in each case alkali treatment brought about an increase in the ε-caprolactone yield, the effect was most pronounced for unground sepiolite, and gradually diminished for the catalysts obtained from milled samples. The results indicated that the MSH phase, developed in alkali-treated ground sepiolite, was catalytically less active than the nanocrystalline Mg(OH)_2_ particles formed in the NaOH-activated parent sepiolite. In view of the very different textural properties of catalysts derived by alkali activation from the as-received sepiolite and from the ground samples it cannot be unequivocally concluded whether lower activity of the latter was due to the lower areal basicity, or to the diffusional limitations within their micropore system. 

In summary, the results presented in this section showed that both grinding and alkali activation are important tools in shaping the basicity of sepiolite-derived catalysts and their performance in base-catalyzed reactions. Short grinding caused an increase of basicity, but upon extended treatment a fall of basicity with respect to the parent sepiolite was observed. In contrast, the NaOH activation always increased the basicity in comparison with the starting material. However, the results of catalytic tests demonstrated that basicity was not the only parameter controlling the course of catalytic reactions over these materials. Factors such as modification of textural properties and/or change of the phase composition occurring upon the above treatments could also influence the catalytic pattern.

Selected sepiolite materials investigated in this work were also subjected to tests of CO_2_ capture and the results are given in Table 4. The untreated sepiolite showed CO_2_-sorption capacity of 1.5 mmolg^−1^, which is in agreement with previous reports [12,67]. This places sepiolite at the top of the list of natural clay mineral sorbents tested for CO_2_ entrapment [68]. CO_2_ sorption by raw sepiolite was attributed primarily to physisorption within the network of structural micropores. Grinding evidently worsened the CO_2_-uptake ability, the effect easily explicable considering the collapse of the porous system in milled sepiolite. The effect of alkali activation on CO_2_-sorption properties differed, depending on the application of grinding pretreatment or lack thereof. This is understandable in view of different nature of phases evolving in both types of materials. Thus, the NaOH-treated, unground sepiolite (Sep/NaOH-24 h), which was a composite of loughlinite and nanocrystalline Mg(OH)_2_, showed a poorer performance than the as-received sepiolite (Sep), due to blocking of micropores by Mg(OH)_2_ particles. In contrast, all tested alkali-treated ground samples (Sep/gr-10’/NaOH-24 h, Sep/gr-30’/NaOH-24 h, and Sep/gr-60’/NaOH-24 h) performed better than the ground materials from which they were derived (Sep/gr-10’, Sep/gr-30’, and Sep/gr-60’). The improvement was especially spectacular for Sep/gr-30’/NaOH-24 h and Sep/gr-60’/NaOH-24 h, in which formation of magnesium-rich MSH phase with a well-developed microporous texture was observed. The maximum value of CO_2_-capture capacity, equal to 1.9 mmolg^−1^ was observed for the Sep/gr-30’/NaOH-24 h sample. The Sep/gr-60’/NaOH-24 h sample displayed a slightly lower capacity of 1.8 mmolg^−1^, possibly due to somewhat lower contribution of micropores in the total porosity. A literature search revealed that, up to now, modification of sepiolite which aimed at boosting its CO_2_-sorption capacity consisted mainly in acid activation, followed by functionalization with various amines [67,69,70,71]. Thus, acid treated sepiolite impregnated with tetraethylenepentamine displayed a CO_2_-sorption capacity of 2.2 mmol/g [69]; with triethylenetetramine 1.9 mmol/g [70]; with polyethyleneimine 1.8 mmol/g [71]; while doubly functionalized by grafting with (3-aminopropyl)triethoxysilane followed by impregnation with polyethyleneimine captured 2.1 mmol/g CO_2_ [67]. This comparison shows that modification of sepiolite described in this work, consisting of grinding and NaOH activation, which is a simpler and cheaper procedure than immobilization of polyamines on acid-treated sepiolite, yields sorbents of comparable CO_2_-uptake capacity. 

## 4. Conclusions

The study revealed that grinding pretreatment of natural sepiolite changed the pathway of phase transformation of this mineral upon treatment with NaOH solution. Thus, wet alkali activation of the as-received sepiolite led, as previously described [12], to the formation of partially desilicated loughlinite (Na-sepiolite) with a pore system blocked by nanocrystalline Mg(OH)_2_. In contrast, the ground sepiolite with strongly amorphized structure was much more susceptible to desilication and transformed into magnesium silicate hydrate phase, with Mg/Si ratio much higher than in the parent clay, and a well-developed microporous texture. The absence of nanocrystalline Mg(OH)_2_ in alkali-treated, shortly ground sepiolite was attributed to its consumption during MSH phase nucleation. On the other hand, in the most strongly ground and desilicated sample, not all Mg could be accommodated by the MSH phase, and the excess formed well crystalline Mg(OH)_2_.

^29^Si MAS NMR analysis identified a different mechanism of desilication in the as-received and the ground sepiolite. In the former, preferential leaching of Si from the center of structural ribbons occurred, in the latter a facile loss of Si from edge positions was additionally observed, pointing to ribbon-ribbon linkages as particularly prone to milling-induced structural damage. 

Results of catalytic tests showed that both grinding and alkali activation are important tools in shaping the basicity of sepiolite-derived catalysts and their performance in base-catalyzed reactions. In all cases treatment with NaOH led to enhancement of basicity with respect to the parent material. The effect of grinding depended on the extent of treatment and caused an increase of basicity for short- and a decrease for long-milling. However, the observed catalytic trends depended not only on surface basicity, but were also influenced by large differences in textural properties and phase composition of the alkali treated products. 

Joint grinding and alkali activation proved a simple and effective method for boosting CO_2_-sorption capacity of sepiolite, and the resulting material performed in a manner comparable to amine functionalized, acid-activated sepiolite sorbents.

## Figures and Tables

**Table 1:** EDX-determined chemical composition of selected samples (wt.% of metal oxides and loss on ignition), Mg/Si atomic ratio in the solids, amount of leached Mg and Si (as per cent of the initial content in the parent sample). (LOI—loss on ignition).

Sample	SiO_2_	MgO	Al_2_O_3_	CaO	Fe_2_O_3_	K_2_O	Na_2_O	LOI	Mg/Si	Mg Loss [%]	Si Loss [%]
Sep	53.1	21.4	1.9	2.2	0.4	0.4	0.1	20.6	0.60	-	-
Sep/NaOH-3 h	49.6	21.9	2.0	2.2	0.4	0.4	3.8	19.7	0.65	0.3	9.1
Sep/NaOH-24 h	46.8	22.6	2.0	2.1	0.4	0.3	3.8	22.0	0.73	0.3	15.9
Sep/gr-10’	54.2	22.0	2.1	2.3	0.4	0.5	0.1	18.6	0.59	-	-
Sep/gr-10’/NaOH-3 h	46.4	22.4	2.2	2.2	0.4	0.5	3.5	22.5	0.72	-	-
Sep/gr-10’/NaOH-24 h	46.0	23.3	2.1	2.2	0.4	0.2	4.0	21.9	0.75	0.2	21.1
Sep/gr-30’	54.5	22.3	2.2	2.1	0.4	0.5	0.1	17.9	0.61	-	-
Sep/gr-30’/NaOH-3 h	46.0	26.1	2.4	2.6	0.5	0.3	2.2	19.9	0.85	-	-
Sep/gr-30’/NaOH-24 h	40.3	25.4	2.9	2.9	0.8	0	2.9	25.0	0.93	0.3	34.1
Sep/gr-60’	55.4	21.8	2.0	2.5	0.4	0.5	0.2	17.3	0.59	-	-
Sep/gr-60’/NaOH-3 h	40.5	27.2	2.3	2.6	0.4	0	1.4	25.6	1.00	-	-
Sep/gr-60’/NaOH-24 h	38.5	28.2	2.5	2.7	0.4	0	1.7	26.0	1.09	0.3	43.8

**Table 2:** Parameters of ^29^Si MAS NMR spectra components obtained from deconvolution.

Sample	^29^Si MAS NMR Parameter	Q^1^	Q^2^	Q^3^	Q^4^
Sep	center (ppm)	-	−85.9	−92.0, −94.5, −98.0	-
	FWHM (ppm)	-	4.9	2.2, 1.9, 3.5	-
	intensity (%)	-	4	34, 30, 32	-
Sep/NaOH-24 h	center (ppm)	-	-	−92.5, −94.7, −97.6	-
	FWHM (ppm)	-	-	3.9, 3.1, 5.1	-
	intensity (%)	-	-	54, 6, 40	-
Sep/gr-10’	center (ppm)	−80.5	−86.2	−91.8, −94.6, −97.9	−106.6
	FWHM (ppm)	10.3	5.7	3.2, 3.1, 5.1	11.2
	intensity (%)	7	13	23, 26, 22	9
Sep/gr-10’/NaOH-24 h	center (ppm)	−79.1	−84.5	−92.8	-
	FWHM (ppm)	5.9	5.1	6.9	-
	intensity (%)	5	17	78	-
Sep/gr-30’	center (ppm)	−80.0	−85.2	−94.4	−106.8
	FWHM (ppm)	10.1	5.5	9.1	11.8
	intensity (%)	9	11	67	13
Sep/gr-30’/NaOH-24 h	center (ppm)	−79.2	−84.5	−92.5	-
	FWHM (ppm)	5.4	4.9	7.6	-
	intensity (%)	9	22	69	-
Sep/gr-60’	center (ppm)	−79.8	−85.3	−94.1	−106.8
	FWHM (ppm)	12.0	6.7	9.5	14.5
	intensity (%)	11	13	55	21
Sep/gr-60’/NaOH-24 h	center (ppm)	−78.4	−84.5	−92.6	-
	FWHM (ppm)	5.9	6.1	7.4	-
	intensity (%)	10	34	56	-

**Table 3:** Textural parameters from N_2_ adsorption/desorption isotherms at −196 °C (S_BET_, V_tot_, S_micro_, V_micro_, D_av_).

Sample	S_BET_ [m^2^g^−1^]	V_tot_ [cm^3^g^−^^1^]	S_micro_ [m^2^g^−1^]	V_micro_ [cm^3^g^−^^1^]	D_av_ [Å]
Sep	299	0.44	140	0.059	59
Sep/NaOH-3 h	108	0.35	12	0.005	131
Sep/NaOH-24 h	119	0.37	20	0.009	124
Sep/gr-10’	182	0.37	85	0.052	82
Sep/gr-10’/NaOH-3 h	228	0.41	67	0.031	73
Sep/gr-10’/NaOH-24 h	223	0.39	54	0.025	69
Sep/gr-30’	70	0.15	30	0.020	88
Sep/gr-30’/NaOH-3 h	266	0.30	63	0.030	45
Sep/gr-30’/NaOH-24 h	320	0.31	91	0.041	39
Sep/gr-60’	31	0.09	10	0.008	115
Sep/gr-60’/NaOH-3 h	304	0.29	83	0.039	38
Sep/gr-60’/NaOH-24 h	328	0.34	63	0.030	41

**Table 4:** Total basicity from benzoic acid titration, yield of DAA obtained in self-condensation of acetone, yield of ε-caprolactone in Baeyer-Villiger oxidation of cyclohexanone, and amount of sorbed CO_2_. Standard error of the mean value in parentheses.

Sample	Basicity [μmolg^−1^]/[μmolm^−2^]	DAA Yield [mmolg^−1^]	ε-Caprolactone Yield [mmolg^−1^]	CO_2_ Sorption [mmolg^−1^]
Sep	214 ± 11 / 0.7	0.44 ± 0.01	21.0 ± 0.6	1.5 ± 0.02
Sep/NaOH-3 h	1039 ± 16 / 9.6	8.64 ± 0.03	36.1 ± 1.0	-
Sep/NaOH-24 h	1090 ± 22 / 9.2	9.02 ± 0.03	36.9 ± 0.7	1.2
Sep/gr-10’	265 ± 8 / 1.5	2.32 ± 0.01	17.2 ± 0.8	1.1
Sep/gr-10’/NaOH-3 h	833 ± 26 / 3.7	12.21 ± 0.03	25.5 ± 0.7	-
Sep/gr-10’/NaOH-24 h	790 ± 3 / 3.5	14.37 ± 0.07	31.9 ± 0.6	1.3
Sep/gr-30’	167 ± 6 / 2.3	0.80 ± 0.01	16.8 ± 0.7	0.9
Sep/gr-30’/NaOH-3 h	983 ± 33 / 3.7	16.83 ± 0.05	23.1 ± 0.6	-
Sep/gr-30’/NaOH-24 h	1133 ± 29 / 3.5	20.62 ± 0.03	29.0 ± 0.9	1.9
Sep/gr-60’	80 ± 4 / 2.9	0.53 ± 0.01	15.7 ± 1.0	0.7
Sep/gr-60’/NaOH-3 h	986 ± 33 / 3.2	18.11 ± 0.04	25.2 ± 0.6	-
Sep/gr-60’/NaOH-24 h	1185 ± 45 / 3.6	17.59 ± 0.05	25.6 ± 0.9	1.8
